# Supplementary material for: The regulatory control of Cebpa enhancers and silencers in the myeloid and red-blood cell lineages
Source: PLoS One. 2019 Jun 10;14(6):e0217580. doi: 10.1371/journal.pone.0217580 (PMC6557489; doi:10.1371/journal.pone.0217580)
Supplement: S1 Text — Supporting figures and tables. (PDF) [file pone.0217580.s001.pdf]

The regulatory control of *Cebpa* enhancers and silencers in the myeloid and red-blood cell lineages

Supporting Information

Andrea Repele, Shawn Krueger, Tapas Bhattacharyya, Michelle Y. Tuineau, & Manu*

Department of Biology,
University of North Dakota,
10 Cornell Street, Stop 9019, Grand Forks, ND 58202-9019, U.S.A.

*Corresponding author

E-mail: manu.manu@und.edu

Contents

Supporting Figures 5

Supporting Tables 18

List of Figures

A Flow cytometry and morphological staging of PUER differentiation. 5
B Immunoblot of C/EBPα protein expression. 6
C Normalization of Firefly luminescence against Renilla luminescence for wildtype enhancers in PUER cells. 7
D Sequence-based model of transcription 8
E Normalization of Firefly luminescence against Renilla luminescence for mutants of *Cebpa(7)*, *Cebpa(14)*, and *Cebpa(16)* in PUER cells. 9
F The regulatory logic of *Cebpa(18)* and the +37kb enhancer. 10
G Normalization of Firefly luminescence against Renilla luminescence for *Cebpa(18m1)* and the +37kb enhancer in uninduced PUER cells. 11
H Relative activity of *Cebpa* silencers in PUER cells. 12
I Normalization of Firefly luminescence against Renilla luminescence for wildtype silencers in PUER cells. 13
J Normalization of Firefly luminescence against Renilla luminescence for wildtype silencers in G1ME cells. 14
K *Cebpa* expression is undetectable in G1ME cells. 15
L Normalization of Firefly luminescence against Renilla luminescence for mutant silencers in G1ME cells. 16
M Compilation of ChIP-seq and ChIP-chip datasets from NCBI Gene Expression Omnibus 17

List of Tables

A Binding sites mutated in the study. 18
B Scheme for the synthesis of mutant CRMs. 19
C Primer and synthetic dsDNA sequences 20

Supporting Figures

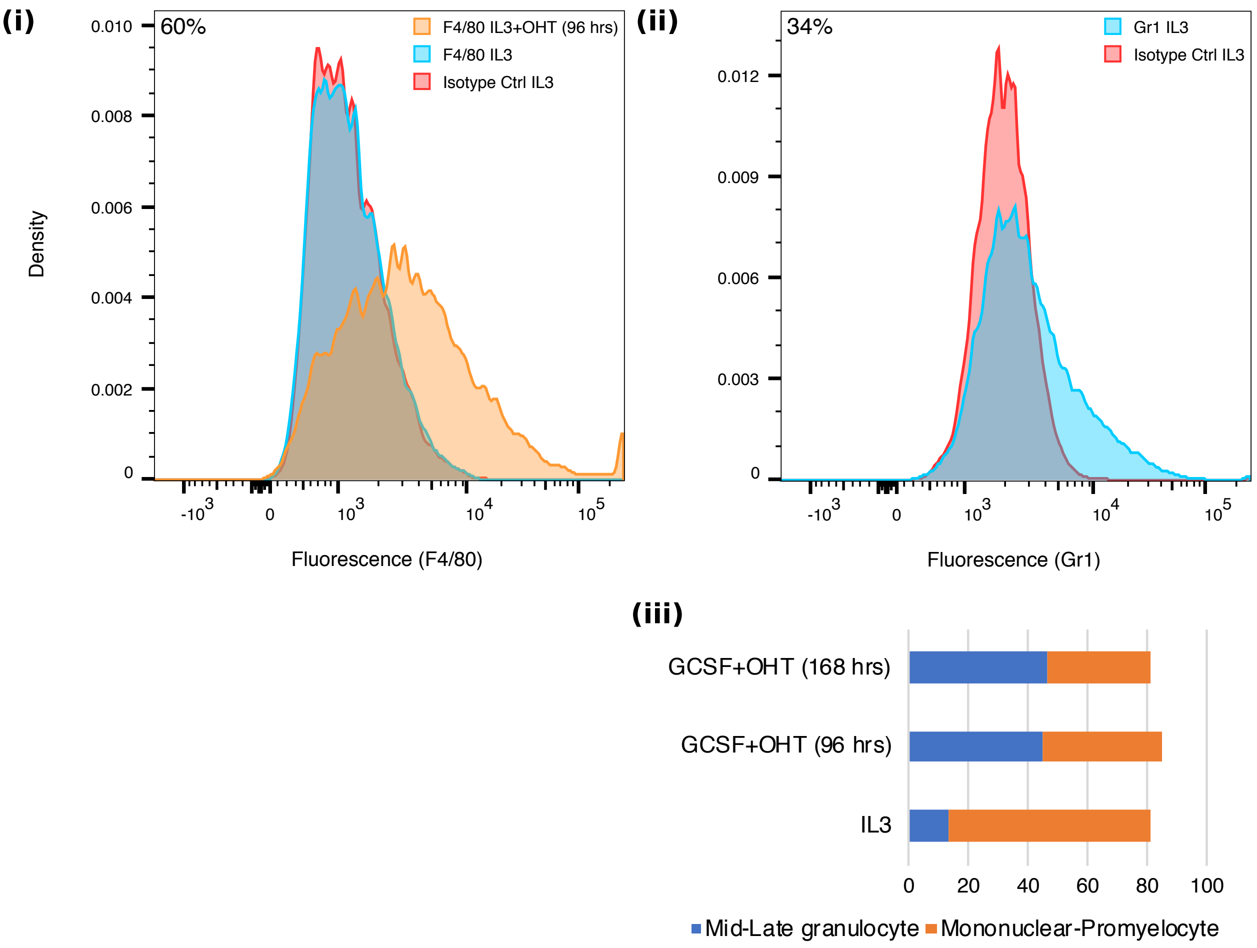

Figure A: Flow cytometry and morphological staging of PUER differentiation. **i**. Histograms of the macrophage marker F4/80 fluorescence in PUER cells with and without OHT treatment. There is no detectable F4/80 expression in undifferentiated PUER cells. F4/80 is upregulated at 96 hours of OHT treatment in IL3 conditions. **ii**. Histograms of the neutrophil marker Gr-1 in undifferentiated PUER cells, showing that they express Gr-1, which is in agreement with previous analyses [1]. As discussed in the study that established PUER neutrophil differentiation [1], Gr-1 is not informative for assessing differentiation since it is already expressed in uninduced PUER cells. We score cells based on their morphology in Wright Giemsa stains to asess neutrophil differentiation. **iii**. Cells were staged into early, middle, and late stages of neutrophil development according to the scheme of Zhou *et al.* [2]. The early stage comprised mononuclear myeloblasts (oval nuclei with high nucleocytoplasmic ratio) and promyelocytes (lower nucleocytoplasmic ratio and beginning of nuclear clearing). The middle stage consists of myelocytes (small clearing in the nucleus) and metamyelocytes (ring shaped nucleus). The late stage was defined by band cells (thin ring-shaped nucleus) and mature neutrophils (curled/ringed or fully segmented nucleus). During the course of OHT treatment in GCSF conditions, the morphological distribution shifts significantly in favor of middle and late stage neutrophils.

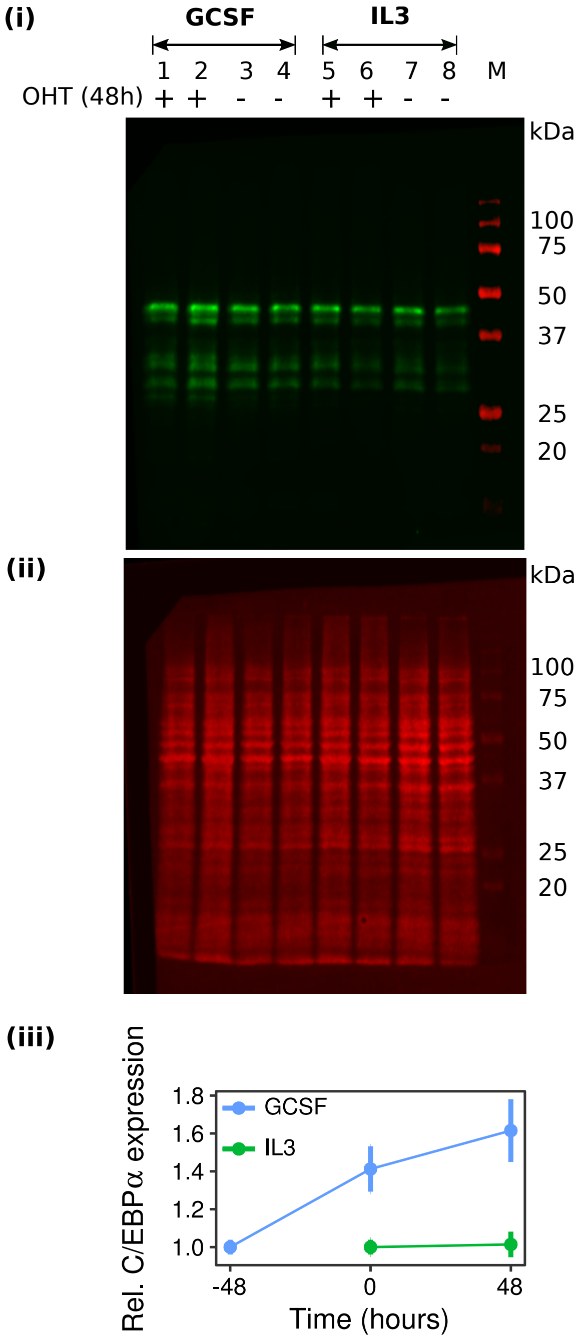

Figure B: Immunoblot of C/EBPα protein expression. **i**. Flourescent detection with anti-C/EBPα in the 800nm channel. Two biological replicates are shown. Lanes 1–4 were treated with GCSF for 48 hours and lanes 1–2 were treated with OHT for an additional 48 hours in the prescence of GCSF. Both the 42kDa and 30kDa isoforms are detected. **ii**. Detection of total protein in the 700nm channel with the REVERT stain (Licor). **iii**. Time course of C/EBPα protein expression mirrors *Cebpa* mRNA expression (Fig. 1C). Band intensities were summed across all bands and normalized against total protein. Relative expression was normalized to average relative expression in uninduced PUER cells. -48 hours and 0 hour points are both measurements from uninduced cells. Error bars show standard error.

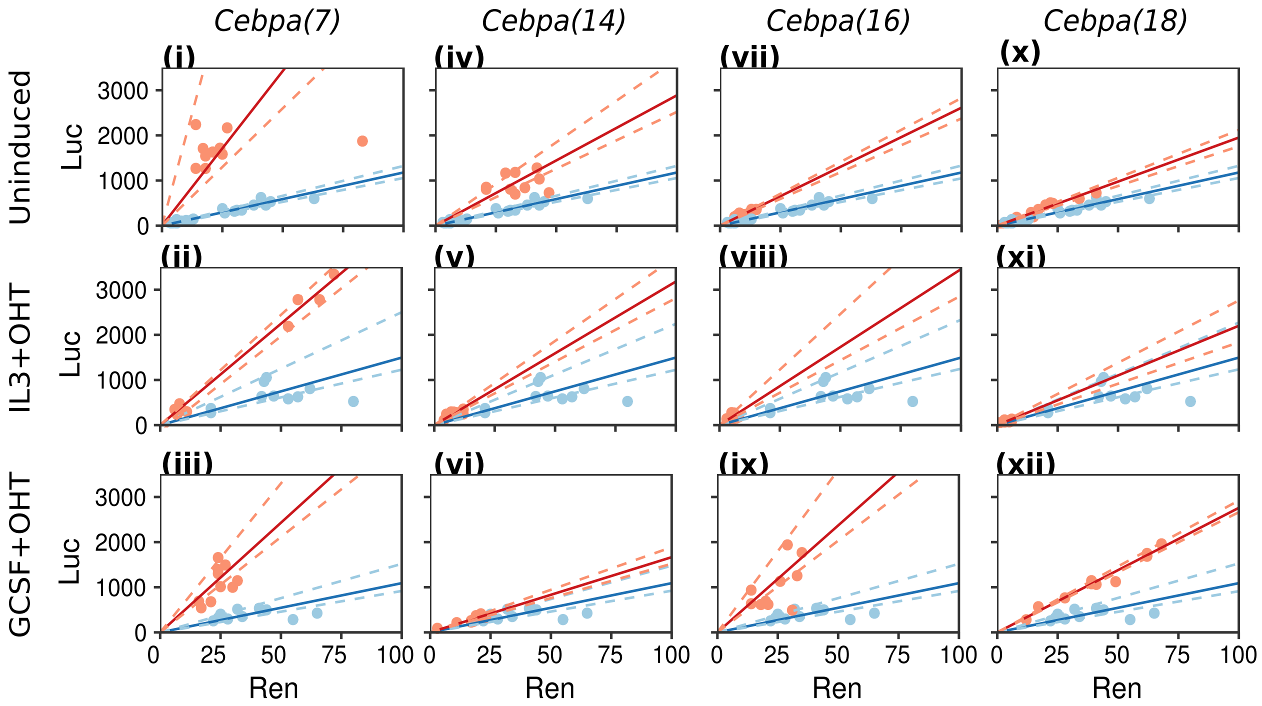

Figure C: Normalization of Firefly luminescence against Renilla luminescence for wildtype enhancers in PUER cells. Scatter plots of Firefly luminescence (y-axis) against Renilla luminescence (x-axis). Blue points show the luminescence measurements of the construct containing the *Cebpa* proximal promoter alone, *Cebpa(0)*, in all panels. Red points are measurements of constructs bearing the indicated CRM in addition to the *Cebpa* proximal promoter. Best fit lines $y = \beta x$ were determined using robust errors-in-variables (EIV) regression and are plotted as solid lines. The normalized activity is given by estimated slope, β. 95% confidence intervals for the slope are shown as dashed lines. **i–iii**. *Cebpa(7)*. **iv–vi**. *Cebpa(14)*. **vii–ix**. *Cebpa(16)*. **x–xii**. *Cebpa(18)*. **i, iv, vii, x**. Uninduced PUER cells in IL3. **ii, v, viii, xi**. PUER cells after 24h OHT treatment in IL3. **iii, vi, ix, xii**. PUER cells after 24h OHT treatment in GCSF.

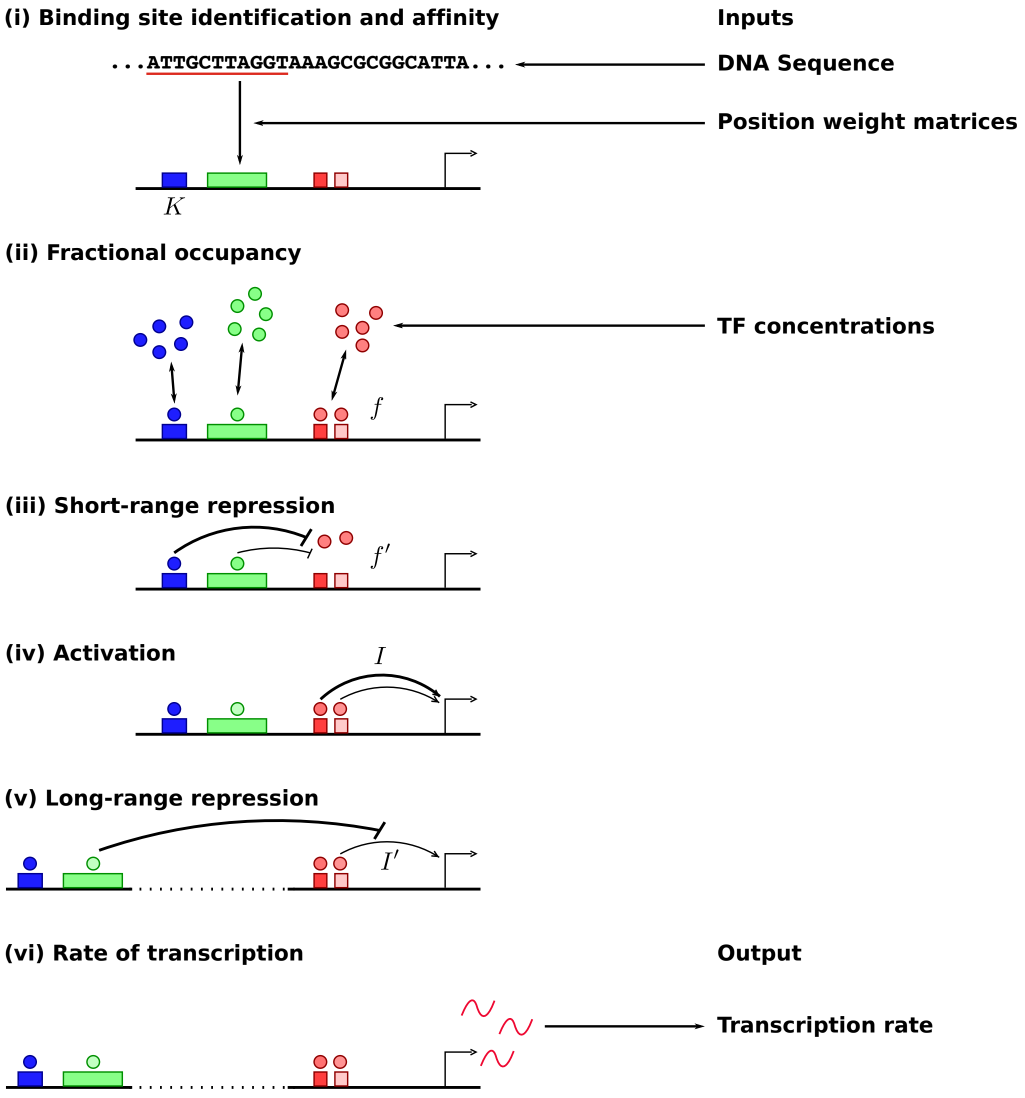

Figure D: Sequence-based model of transcription. See Bertolino *et al.* for a detailed description and equations. **i**. Binding sites are identified and their binding affinities (K) are computed from CRM and promoter DNA sequences using PWMs. Activator and repressors sites are shown in red and green/blue respectively. **ii**. Binding of TFs to their sites is simulated using thermodynamic principles to compute the fractional occupancy (f) of each site based on the concentrations of the TFs and binding site affinities. **iii**. Quenching, or short-range repression, is simulated by reducing the occupancy of activators (f') based on the occupancy of repressors bound within 150bp. **iv**. The interaction strength (I) of the CRM with the basal promoter is determined based on the occupancy of the activator sites and the activation efficiency of the bound TFs. **v**. Long-distance dominant repression is simulated by reducing the interaction strength (I') based on the occupancy of the repressors bound to the CRM. **vi**. In the last step the rate of transcription is computed by modeling transcript initiation as an enzymatic reaction, where the activation energy barrier is lowered in proportion to the interaction strength (I').

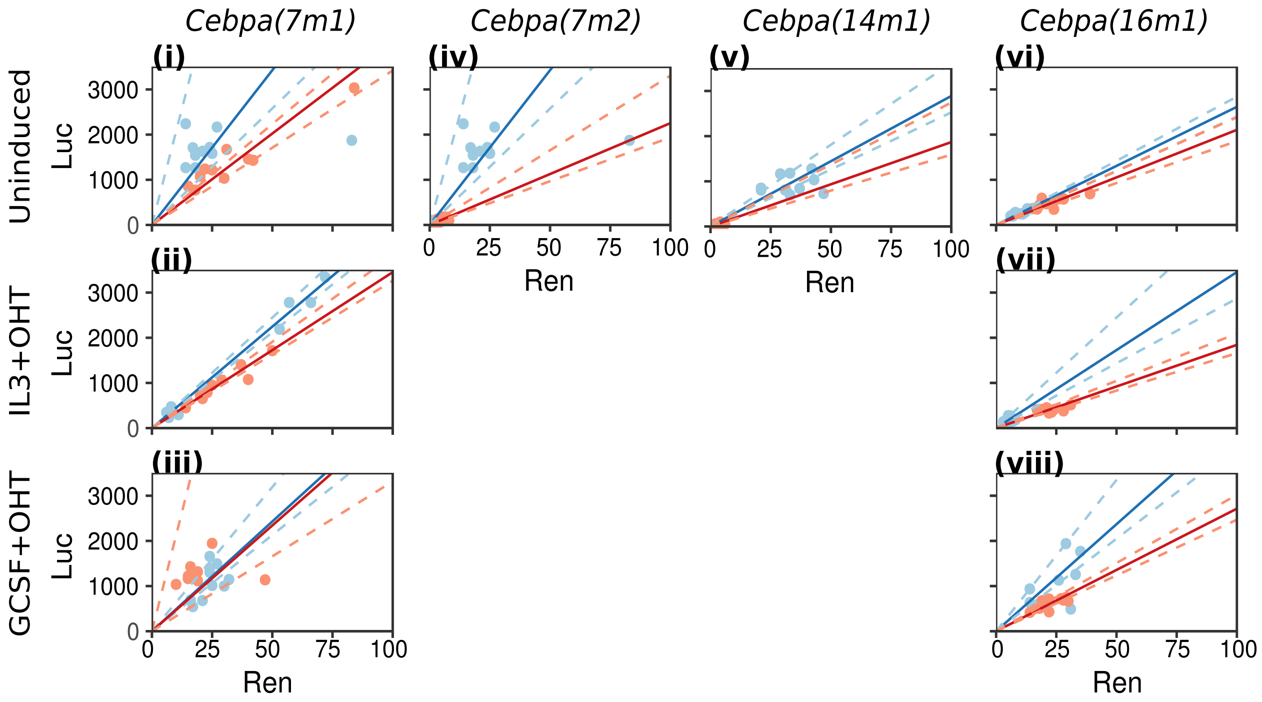

Figure E: Normalization of Firefly luminescence against Renilla luminescence for mutants of *Cebpa(7)*, *Cebpa(14)*, and *Cebpa(16)* in PUER cells. See legend of Figure C for details of the calculations, axes, and legends. **i–iii**. *Cebpa(7m1)* (red) and *Cebpa(7)* (blue). **iv**. *Cebpa(7m2* (red) and *Cebpa(7)* (blue). **v**. *Cebpa(14m1)* (red) and *Cebpa(14)* (blue). **vi–viii**. *Cebpa(16m1)* (red) and *Cebpa(16)* (blue). **i, iv, v, vi**. Uninduced PUER cells in IL3. **ii, vii**. PUER cells after 24h OHT treatment in IL3. **iii, viii**. PUER cells after 24h OHT treatment in GCSF.

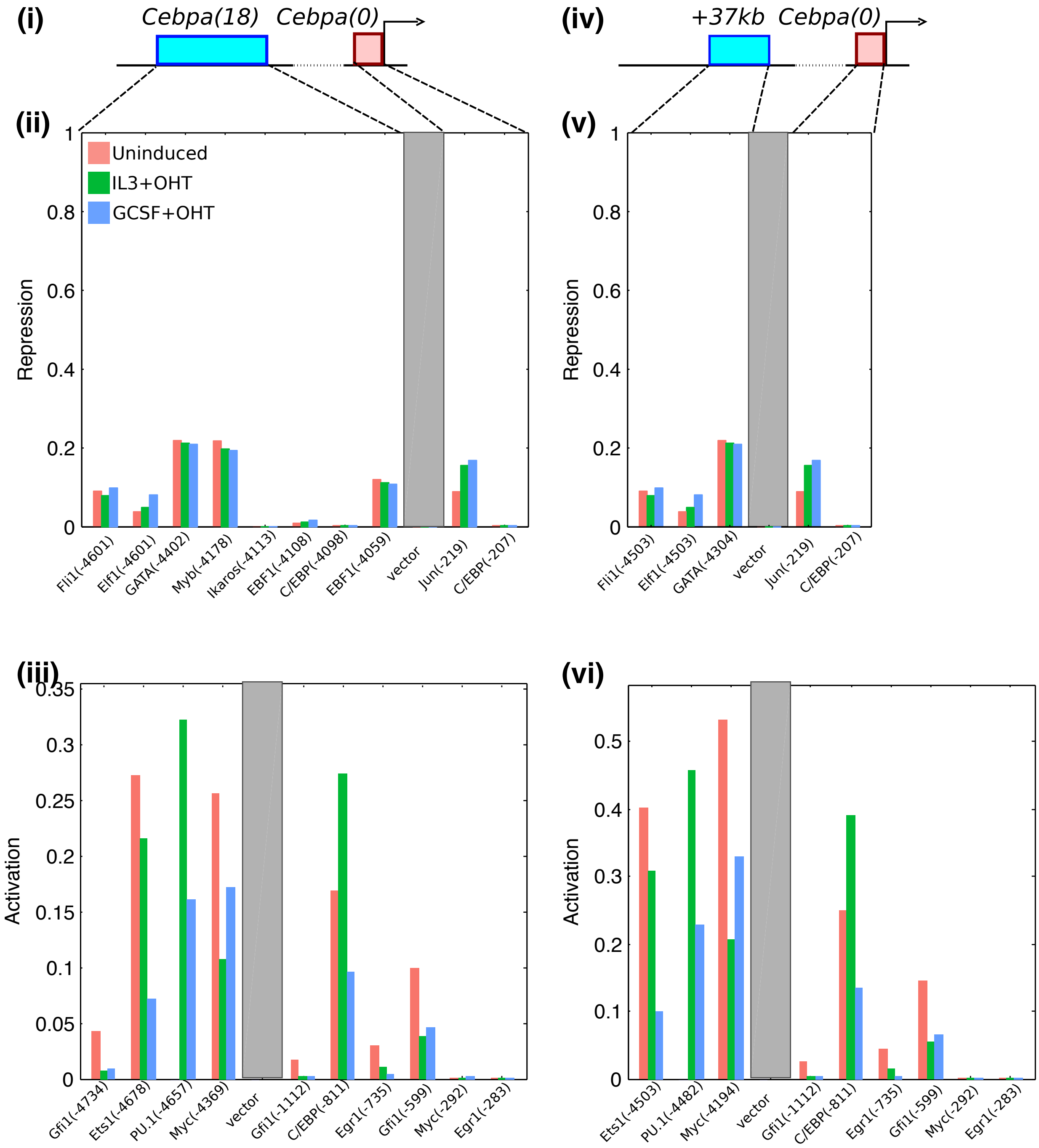

Figure F: The regulatory logic of *Cebpa(18)* and the +37kb enhancer. **i–iii**. *Cebpa(18)*. **iv–vi**. The +37kb enhancer [3]. **i, iv**. Schematics of the construct design showing distal CRM (blue) and *Cebpa* proximal promoter (red). **ii, v**. Activity of each TF repressor site predicted by the sequence-based model. See the legend of Figure 4F for details of the calculations, axes, and legend. **iii, vi**. Activity of each TF activator site predicted by the sequence-based model. See the legend of Figure 3B,F for details of the calculations, axes, and legend.

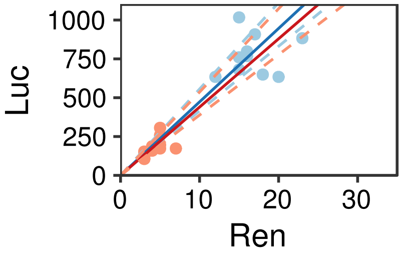

Figure G: Normalization of Firefly luminescence against Renilla luminescence for *Cebpa(18m1)* and the +37kb enhancer in uninduced PUER cells. See legend of Figure C for details of the calculations, axes, and legends. Blue and red points show the luminescence measurements of *Cebpa(18m1)* and the +37kb enhancer respectively in uninduced PUER cells.

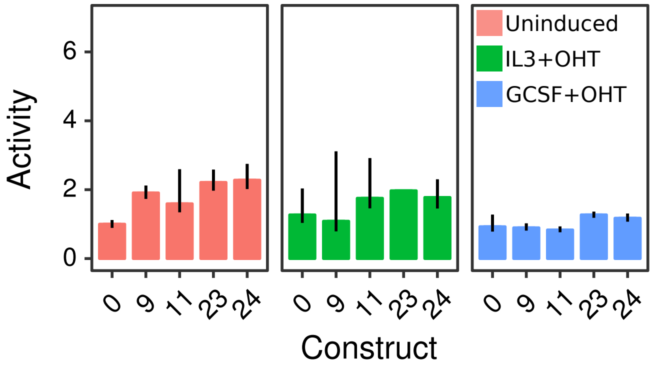

Figure H: Relative activity of *Cebpa* silencers in PUER cells. *Cebpa(0)* is the construct bearing the *Cebpa* proximal promoter alone, while the others carry the indicated distal CRM in addition to the proximal promoter. Bar plots show the ratio of each construct's activity in each condition to *Cebpa(0)* activity in uninduced PUER cells. Each CRM's activity was assayed in uninduced (red), induced IL3 (green), induced GCSF (blue) conditions. Reporter assays were performed in 10 replicates. Error bars are 95% confidence intervals. Regression plots are shown in Figure I.

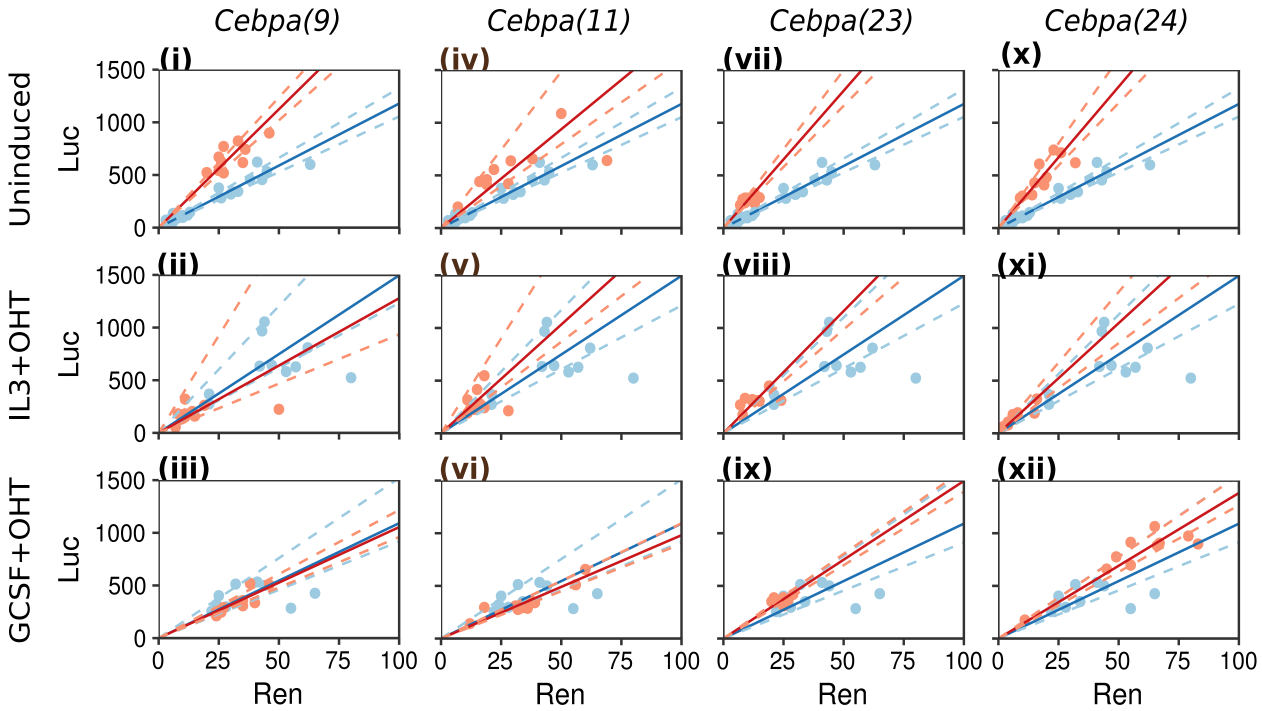

Figure I: Normalization of Firefly luminescence against Renilla luminescence for wildtype silencers in PUER cells. See legend of Figure C for details of the calculations, axes, and legends. Blue points show the luminescence measurements of the construct containing the *Cebpa* proximal promoter alone, *Cebpa(0)*, in all panels. **i–iii**. *Cebpa(9)*. **iv–vi**. *Cebpa(11)*. **vii–ix**. *Cebpa(23)*. **x–xii**. *Cebpa(24)*. **i, iv, vii, x**. Uninduced PUER cells in IL3. **ii, v, viii, xi**. PUER cells after 24h OHT treatment in IL3. **iii, vi, ix, xii**. PUER cells after 24h OHT treatment in GCSF.

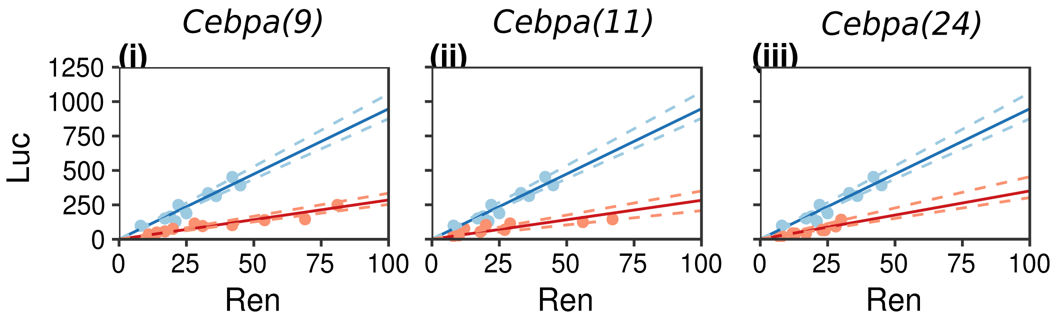

Figure J: Normalization of Firefly luminescence against Renilla luminescence for wildtype silencers in G1ME cells. See legend of Figure C for details of the calculations, axes, and legends. Blue points show the luminescence measurements of the construct containing the *Cebpa* proximal promoter alone, *Cebpa(0)*, in all panels. Red points are measurements of constructs bearing the indicated CRM in addition to the *Cebpa* proximal promoter. **i**. *Cebpa(9)*. **ii**. *Cebpa(11)*. **iii**. *Cebpa(24)*.

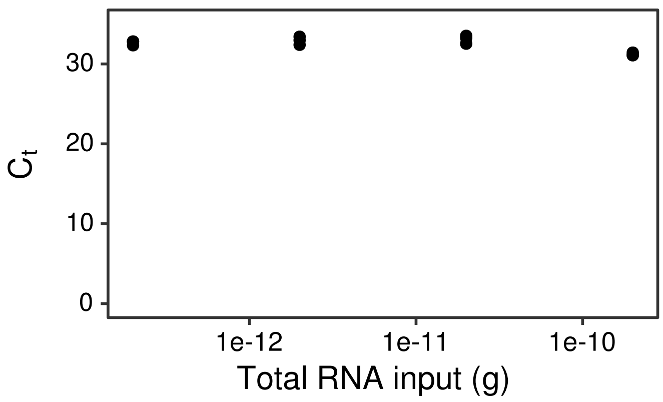

Figure K: *Cebpa* expression is undetectable in G1ME cells. RT-RTPCR of G1ME total RNA with *Cebpa* primers (see Methods). The x-axis is the amount of RNA that was reverse transcribed. The y-axis is the cycle where the fluorescence crosses the threshold (C_t). There is very little change in C_t over three orders of magnitude of total RNA, implying that the sample lacks *Cebpa* mRNA.

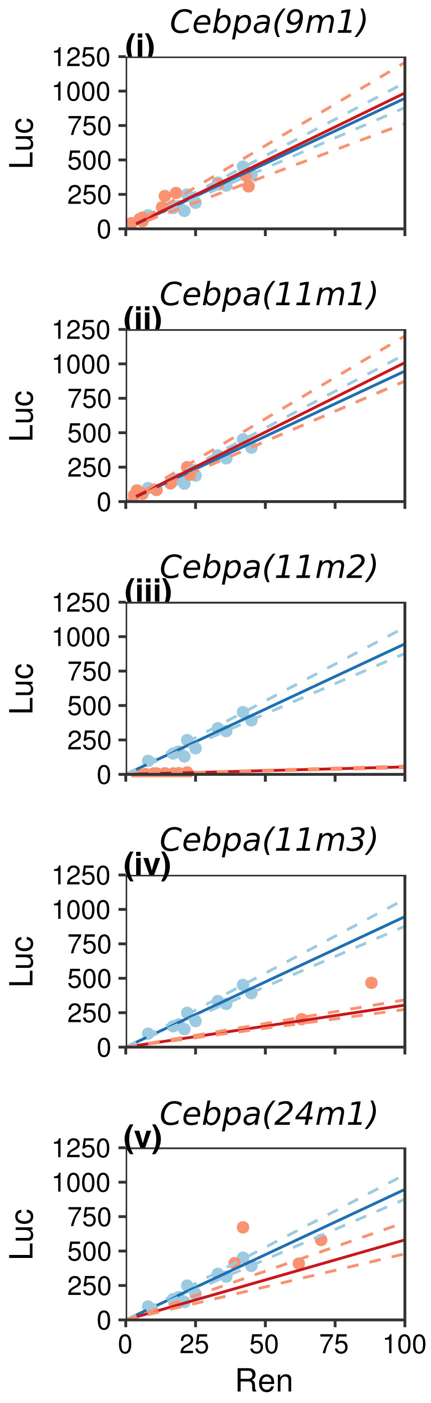

Figure L: Normalization of Firefly luminescence against Renilla luminescence for mutant silencers in G1ME cells. See legend of Figure C for details of the calculations, axes, and legends. Blue points show the luminescence measurements of the construct containing the *Cebpa* proximal promoter alone, *Cebpa(0)*, in all panels. Red points are measurements of constructs bearing the indicated CRM in addition to the *Cebpa* proximal promoter. **i**. *Cebpa(9m1)*. **ii**. *Cebpa(11m1)*. **iii**. *Cebpa(11m2)*. **iv**. *Cebpa(11m3)*. **v**. *Cebpa(24m1)*.

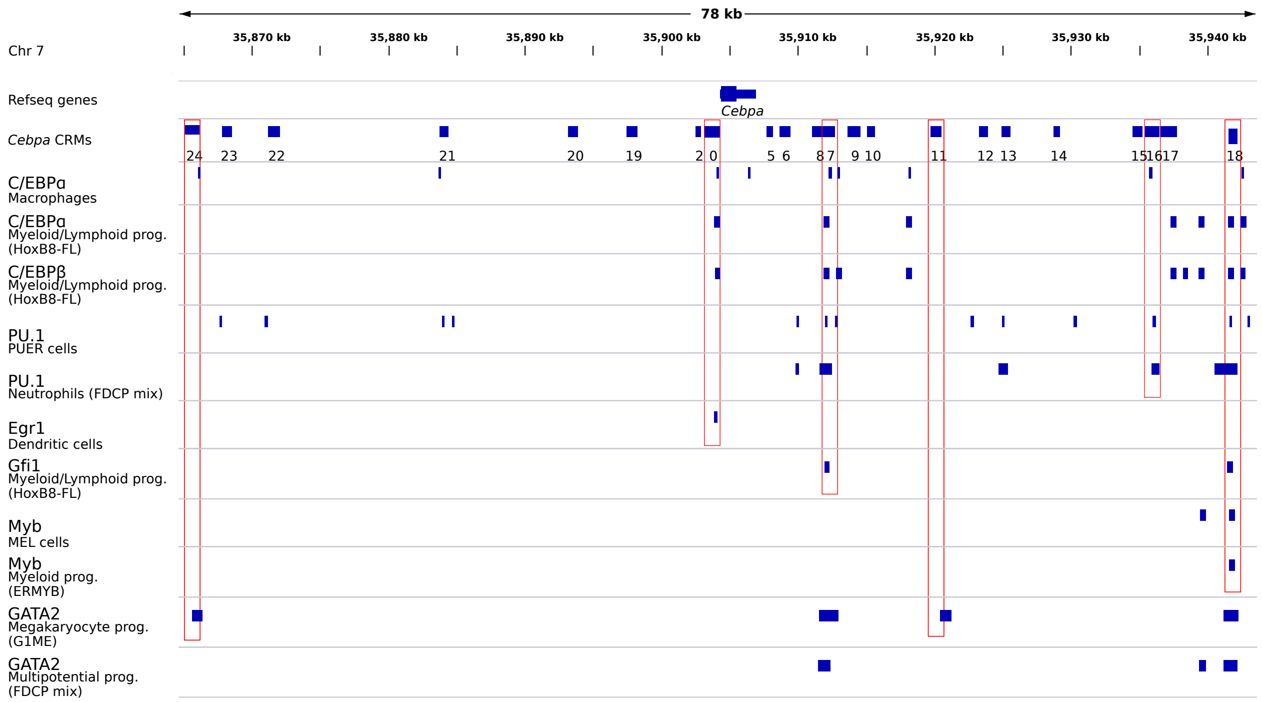

Figure M: Compilation of ChIP-seq and ChIP-chip datasets from NCBI Gene Expression Omnibus. Where available, BED format files were downloaded and plotted in Integrated Genomics Viewer [4]. The first track shows annotated genes in the genomic region. The second track shows the CRMs analyzed in this study. The other tracks show TF binding peaks from ChIP-seq or ChIP-chip datasets. The TF and the cell type the ChIP was performed in are listed on the left of each track. Empirical evidence for binding is matched with CRMs predicted to be bound by the TF in the red boxes. Tracks 3-13: GSM537984 [5], GSM2231898 [6], GSM2231899 [6], GSM538003 [5], GSM1218228 [7], GSM881139 [8], GSM2231903 [6], GSM912903 (mouse ENCODE), GSE22095 [9], GSM777091 [10, 11], and GSM1218221 [7].

Supporting Tables

Construct	TF	PWM Name	Source	Accession	PWM min score	PWM max score	Position	WT sequence	WT score	Mutant sequence	Mutant score
Cebpa(7)	Gfi1	GFI1_01	TRANSFAC	M00250	-34.878	15.686	-4421	GCATGACAAATCACTCTCAGCACA	7.85	gtgtttgtccaagtcaagacctga	-17.30
Cebpa(7)	C/EBP family	CEBPD_Q6	TRANSFAC	M00621	-30.150	10.778	-4385	TGTTAAGAAATG	6.96	tgaccacaggag	-13.64
Cebpa(7)	C/EBP family	CEBPD_Q6	TRANSFAC	M00621	-30.150	10.778	-4372	GTTTGCCTCACT	7.06	tcaacgcaggag	-30.15
Cebpa(7)	Gfi1	GFI1_01	TRANSFAC	M00250	-34.878	15.686	-4244	CACATTCCCACTGATTTATAGGGA	7.70	gtgtttgtccaagtcaagacctga	-12.73
Cebpa(7)	Gfi1	GFI1_01	TRANSFAC	M00250	-34.878	15.686	-4098	CTCACAAGTTCTGATTCCCTGGGG	7.16	gtgtttgtccaagtcaagacctga	-12.73
Cebpa(14)	Egr1	KROX_Q6	TRANSFAC	M00982	-30.794	16.917	-4275	GGGGGGGGGGAGCC	10.06	aaccgtccatgctt	-30.75
Cebpa(14)	Egr1	KROX_Q6	TRANSFAC	M00982	-30.794	16.917	-4104	GGGGTGGGGGCGGG	15.92	atccgactacgctt	-30.75
Cebpa(16)	PU.1	PU1_01	TRANSFAC	M01203	-36.159	14.409	-4517	GAGAAGAGGAAGTCCTG	9.64	cccgcttccgcaacaac	-25.70
Cebpa(16)	PU.1	PU1_01	TRANSFAC	M01203	-36.159	14.409	-4488	AGAAACAGGAACCTGGG	9.41	cccccatccttaacaac	-25.17
Cebpa(16)	PU.1	PU1_01	TRANSFAC	M01203	-36.159	14.409	-4456	GGGAACGGGAAGGGGGC	8.45	cccccgttcaccaacaa	-25.70
Cebpa(18)	Myb	Myb_JASPAR	JASPAR	MA0057.1	-24.891	9.255	-4178	GGCTGTTG	6.91	ccttcgac	-24.79
Cebpa(9)	GATA	GATA3_02	TRANSFAC	M00350	-22.332	9.550	-4985	GCAGATAAAA	6.27	ttgcgaggac	-22.33
Cebpa(9)	Myb	Myb_JASPAR	JASPAR	MA0057.1	-24.891	9.255	-4576	TGCAGTTG	6.67	ccttcgcc	-24.79
Cebpa(11)	EBF1	COE1_Q6	TRANSFAC	M01871	-28.760	12.724	-4834	CCAGTCCCCTGGGG	10.45	gatcgttaatgaga	-8.08
Cebpa(11)	Myb	Myb_JASPAR	JASPAR	MA0057.1	-24.891	9.255	-4711	CAACTGTG	6.32	ttccaagt	-15.89
Cebpa(11)	GATA	GATA3_02	TRANSFAC	M00350	-22.332	9.550	-4670	AGAGATAATT	7.54	ccgacaggat	-20.36
Cebpa(24)	Myb	Myb_JASPAR	JASPAR	MA0057.1	-24.891	9.255	-4814	GACTGTTG	6.45	ccttcgac	-24.79
Cebpa(24)	GATA	GATA3_02	TRANSFAC	M00350	-22.332	9.550	-4287	ACAGATAATA	7.51	ttgccgggac	-22.33

Table A: Binding sites mutated in the study.

Mutant	Fragments
Cebpa(7m1)	7Amp1+7Amp2
Cebpa(7m2)	7sdsDNA
Cebpa(14m1)	14sdsDNA
Cebpa(16m1)	16Amp1+16sdsDNA+16Amp2
Cebpa(18m1)	18Amp1+18Amp2
Cebpa(9m1)	9Amp1+9sdsDNA+9Amp2
Cebpa(11m1)	11Amp1+11sdsDNA+11Amp2
Cebpa(11m2)	11Amp3+11Amp4
Cebpa(11m3)	11Amp5+11Amp6
Cebpa(24m1)	24Amp1+24Amp2+24Amp3

Table B: Scheme for the synthesis of mutant CRMs. The second column shows the fragments used to stitch the mutant CRM in the order of their appearance. Amp: amplicon. sdsDNA: synthetic dsDNA. The sequences of synthetic dsDNAs and primers of the amplicons are listed in Table 3.

Fragment	Type	Sequence
Cebpa(0)	Primer fwd	TGGCCTAACTGGCCGGTACCTGAGCTCGCTAGCCTCGAGAACTCCTACCCACAGCCGCG
Cebpa(0)	Primer rev	TCCATGGTGGCTTTACCAACAGTACCGGATTGCCAAGCTTCAGCTTCGGGTCGCGAATG
Cebpa(7)	Primer fwd	AACCTCTACAAATGTGGTAAAATCGATAAGGATCCCCCACTTCCACCCCCTAAGA
Cebpa(7)	Primer rev	GCTGACTGGGTTGAAGGCTCTCAAGGGCATCGGTCGACCCTGAGCAGAGCAACCTTAACA
Cebpa(14)	Primer fwd	AACCTCTACAAATGTGGTAAAATCGATAAGGATCCGTAAGGAATCACAGGGGTCAGT
Cebpa(14)	Primer rev	GCTGACTGGGTTGAAGGCTCTCAAGGGCATCGGTCGACTAGGGTGTTTCAGAAAGTCAGTGT
Cebpa(16)	Primer fwd	AACCTCTACAAATGTGGTAAAATCGATAAGGATCCAAAATCAGTTTATCCCTATGCTGCC
Cebpa(16)	Primer rev	GCTGACTGGGTTGAAGGCTCTCAAGGGCATCGGTCGACTGGGCTGAGGACAACTCTGTGT
Cebpa(18)	Primer fwd	AACCTCTACAAATGTGGTAAAATCGATAAGGATCCATGCCACCCCTCTGATTTTGC
Cebpa(18)	Primer rev	GCTGACTGGGTTGAAGGCTCTCAAGGGCATCGGTCGACCACTGAGTCCCCTGGAATAGA
Cebpa(9)	Primer fwd	AACCTCTACAAATGTGGTAAAATCGATAAGGATCCCCCTGTGGAAGAGTTGGTCA
Cebpa(9)	Primer rev	GCTGACTGGGTTGAAGGCTCTCAAGGGCATCGGTCGACCTAGCCCATTGGTCCTACAAAG
Cebpa(11)	Primer fwd	AACCTCTACAAATGTGGTAAAATCGATAAGGATCCGGGAGGAATAGAGAATTGAGATC
Cebpa(11)	Primer rev	GCTGACTGGGTTGAAGGCTCTCAAGGGCATCGGTCGACCTCTCTGAGCCATCTGCAGT
Cebpa(23)	Primer fwd	AACCTCTACAAATGTGGTAAAATCGATAAGGATCCCTCTTCCCCTAGGCATCTACAA
Cebpa(23)	Primer rev	GCTGACTGGGTTGAAGGCTCTCAAGGGCATCGGTCGACACACAGACACATACCCCCATG
Cebpa(24)	Primer fwd	AACCTCTACAAATGTGGTAAAATCGATAAGGATCCCAGCAGCTTTCTATCAACTTGTG
Cebpa(24)	Primer rev	GCTGACTGGGTTGAAGGCTCTCAAGGGCATCGGTCGACAATGGCAGTTTCTTTCCTGTAGTTC
7Amp1	Primer fwd	AACCTCTACAAATGTGGTAAAATCGATAAGGATCCCCCACTTCCACCCCCTAAGA
7Amp1	Primer rev	gttgaActcctgtggtcaGCGAGCAGACACTGTGCTGAGAGTGATTTGTCATGCTTAGTC
7Amp2	Primer fwd	CTGCTCGCtgaccacaggagTtcaacgcaggagGTTTTGCCTGGTGCGGCAACATTTTAA
7Amp2	Primer rev	GCTGACTGGGTTGAAGGCTCTCAAGGGCATCGGTCGACCCTGAGCAGAGCAACCTTAACA
7sdsDNA	gblock	AACCTCTACAAATGTGGTAAAATCGATAAGGATCCCCCACTTCCACCCCCTAAGAATACTGGATCCCTCTTGCCGATAAGGAACTGTGGTCAACTTCTAGTGGCTTTCCT
		GTGCACGTGTTGGGCAACCAAGCCTCAGCTGGACTTAGTTGCCAAGCCCAGACAACAGGTGGCAAGGGGGTGTCAGGGACTGGGTACCAGCTCTTTGGGGAGCTGCCATG
		ACCTTCACCATCAGGTTAGGACCCGTCAGAAGTGGCCTCCTTGAGTGATTTACAATTTGCAAACATGTTTTATTTGATTCCCGAGTTCTGCCGGGGCAATTACAGTGACT
		AAgtgtttgtccaagtcaagacctgaGTGTCTGCTCGCtgaccacaggagTtcaacgcaggagGTTTTGCCTGGTGCGGCAACATTTTAAAAATAGACTCGCTCACTGTA
		CGCGAAGGCAATTTGTTCCAAATTTTCCCACTAATTTGATTTTAATCTGATATTTAAAATTCGTGTGACgtgtttgtccaagtcaagacctgaATAAGCCCTACCTGGCG
		GCACTGTAATTGGCTTTGGCCCAGGAGTCCACAGGACAGAGCATTTATCCCAGAACAATTTGAAGGCACTCATGTCTTAATGTTTTAAATATAGCCTAATTTAGCgtgtt
		tgtccaagtcaagacctgaGCAGGACAATGAGTGTTAAGGTTGCTCTGCTCAGGGTCGACCGATGCCCTTGAGAGCCTTCAACCCAGTCAGC
14sdsDNA	genestring	AACCTCTACAAATGTGGTAAAATCGATAAGGATCCGTAAGGAATCACAGGGGTCAGTCAGGGCTTCCCTAACTGGAAAACCCAAGTTCAGAGGTACCACAGACTATGACT
		GGGGTTAGAGTTGGAACATGGGGTAGGCCGACCTGGGGTACAAGGGAGGAGGACCCCCAGTGTTCATACCATAGGGGCACCTGCTTCTGCTAGACAGTGaaccgtccatg
		cttTGAGCCATGAGAGAGCACAAGGGAGGTCAAGGGGCAGAAAGGCCAGAGGGTGTCAGCAGGCTCCAGCAGGCTGTGGACACTTGGCCAGAAAGGCCTGTTTACTGAGA
		GGCCTGGGAGGTCAAGGCCCAGGCCTGGAGTTAATCATTAATGGCTCACCCTGCTCGTGGCTGCCTAGTGTGGTCTGGACCAGGCCCCAGTACACAGGTACTGCCCCACT
		GCCACGCTGTGTGTATGatccgactacgcttCAGGGGCAGTACTGGTGTGCTTTGGAGACACTGACTTTCTGAAACACCCTAGTCGACCGATGCCCTTGAGAGCCTTCAA
		CCCAGTCAGC
16Amp1	Primer fwd	AACCTCTACAAATGTGGTAAAATCGATAAGGATCCAAAATCAGTTTATCCCTATGCTGCC
16Amp1	Primer rev	AGCATCTACACCAAATCCCGATGCTATCTATGGTCCTCTGTGCATCTGGACGTGCCATGT
16Amp2	Primer fwd	ACGTGGAAAAACATCACGGATCAAAATGGCTTCCTGGTCTGAAGTGGAGGCATGGTGTGT
16Amp2	Primer rev	GCTGACTGGGTTGAAGGCTCTCAAGGGCATCGGTCGACTGGGCTGAGGACAACTCTGTGT
16sdsDNA	gblock	AGACCAGGAAGCCATTTTGATCCGTGATGTTTTTCCACGTAATAACGGCCCCAAAACATTTTTCCACGTAATAACGGGGGATATGGATAGTTTTTCCACGTAATAACCAC
		ACCCAGCAGATGCAGCCAGAACACCCCAGCCTCCCCTGGCCCCACATTAGCAGGCAGGAGTTAGCATCTACACCAAATCCCGATGCTATCTATGGTCCTCTG
18Amp1	Primer fwd	TTGAGCGAGGCTCTCTGTGTGGCTGGGccttcgacAGACATCTGGTAACCTTTGGGTCCC
18Amp1	Primer rev	GCTGACTGGGTTGAAGGCTCTCAAGGGCATCGGTCGACCACTGAGTCCCCTGGAATAGA
18Amp2	Primer fwd	AACCTCTACAAATGTGGTAAAATCGATAAGGATCCATGCCACCCCTCTGATTTTGC
18Amp2	Primer rev	TTCCATGGGACCCAAAGGTTACCAGATGTCTgtcgaaggCCCAGCCACACAGAGAGCCTC
9Amp1	Primer fwd	AACCTCTACAAATGTGGTAAAATCGATAAGGATCCCCCTGTGGAAGAGTTGGTCA
9Amp1	Primer rev	GTTCTAGGGAAGTTTGGTTAAGAACTCCgtcctcgcaaTATGTGCCTATTCTCCAGGACC
9Amp2	Primer fwd	GGTGCTGGCGTGGGGGCAGGAGCACACCACAccttcgccCACGGGGGACTGAATCTGAG
9Amp2	Primer rev	GCTGACTGGGTTGAAGGCTCTCAAGGGCATCGGTCGACCTAGCCCATTGGTCCTACAAAG
9sdsDNA	genestring	ttgcgaggacGGAGTTCTTAACCAAACTTCCCTAGAACGGAGGGAGCTAACAAGAAAGAACTTTGGAAATCTACCCTCCTCTTTCCCTGTCACTGCCAGGAATGTCACCA
		TGAGAGCAGTTTCAGTTAATGAGCAAACTCCTCAGACAAGGCAGGAAGGCAGCTCTTGGGCCTCACTGTCAAGCACAGGAAGCGACTGGATTCCACTTGCCCGGTGTAGG
		GATGACAGCAGGTATTGAGTGGGACTGCAGGCCTGACATCCTTAGCTCCTCCACACCCAGGACAGCCCGGCTGTCAGCACAGGGCAGCAGAAAGGACAGGGGACAAGCTC
		CAGGTGTGGGCGAGTCCCAGAGCAGCCCGGGGAGAGTGTCACTGTGTGGGTGCTGGCGTGGGGGCAGGAGCACACCACAccttcgcc
11Amp1	Primer fwd	AATTCCAGGCTGGAGATGGGTGGGGAAGGTGGTTTGTAATCTTTATTCAGACTCGGCCCC
11Amp1	Primer rev	GCTGACTGGGTTGAAGGCTCTCAAGGGCATCGGTCGACCTCTCTGAGCCATCTGCAGT
11Amp2	Primer fwd	AACCTCTACAAATGTGGTAAAATCGATAAGGATCCGGGAGGAATAGAGAATTGAGATC
11Amp2	Primer rev	GACAACACCCCGTTTGGCTGTCTCATTAACGATCCCCCCCGCCAGGATTCCCAGACAGAT
11sdsDNA	gblock	GGGGGGGATCGTTAATGAGACAGCCAAACGGGGTGTTGTCAGCCCACATGAAGGCCCTGCTGCTGGCCACATTCTGTAAACAAACATCCACATGTGTGCGCATAGCAACC
		TAGTGCCAAAGGACAAACATTCCAAGTACAAGTTTATGGAGCTGTAGTATCGGGGAGGACCCGACAGGATAGGCAAATCATAAATGCATGTAGTGATGCCTTTTGTCTGT
		GCGAACCCTGCCTGTCTGTTGGGAATTCCAGGCTGGAGATGGGTGGGGAAGGTGGTTTGTAAT
11Amp3	Primer fwd	ttgttgggacAGGCAAATCATAAATGCATGTAGTGATGCCTTTTGTCTGTGCGAACCCTG
11Amp3	Primer rev	GCTGACTGGGTTGAAGGCTCTCAAGGGCATCGGTCGACCTCTCTGAGCCATCTGCAGT
11Amp4	Primer fwd	AACCTCTACAAATGTGGTAAAATCGATAAGGATCCGGGAGGAATAGAGAATTGAGATC
11Amp4	Primer rev	ACATGCATTTATGATTTGCCTgtcccaacaaGTCCTCCCCGATACTACAGCTCCATAAAC
11Amp5	Primer fwd	AGCAACCTAGTGCCAAAGGACAAACAttcctgatACAAGTTTATGGAGCTGTAGTATCGG
11Amp5	Primer rev	GCTGACTGGGTTGAAGGCTCTCAAGGGCATCGGTCGACCTCTCTGAGCCATCTGCAGT
11Amp6	Primer fwd	AACCTCTACAAATGTGGTAAAATCGATAAGGATCCGGGAGGAATAGAGAATTGAGATC
11Amp6	Primer rev	TCCCCGATACTACAGCTCCATAAACTTGTatcaggaaTGTTTGTCCTTTGGCACTAGGTT
24Amp1	Primer fwd	TAGGGCCAACCTCAATGTTTTGAAGGTGTccttcgacTGGGAGGAGGCAGATAATTAGTT
24Amp1	Primer rev	CTTTCCTGCAGTTTGGCTCAACAACTGAGAgtcccggcaaAAAGTTGTGCTGCGCCTGGT

24Amp2	Primer fwd	AACCTCTACAAATGTGGTAAAATCGATAAGGATCCCAGCAGCTTTCTATCAACTTGTG
24Amp2	Primer rev	CTAATTATCTGCCTCCTCCCAgtcgaaggACACCTTCAAAACATTGAGGTTGGCCCTAAT
24Amp3	Primer fwd	GGACCAGGCGCAGCACAACTTTttgccgggacTCTCAGTTGTTGAGCCAAACTGCAGGAA
24Amp3	Primer rev	GCTGACTGGGTTGAAGGCTCTCAAGGGCATCGGTCGACAATGGCAGTTTCTTTCCTGTAGTTC

Table C: Primer and synthetic dsDNA sequences. gblock and genestring are synthetic dsDNAs made by Integrated DNA Technologies or Thermo Fisher Scientific respectively. Amp: amplicon. sdsDNA: synthetic dsDNA.

References

[1] Dahl R, Walsh JC, Lancki D, Laslo P, Iyer SR, Singh H, et al. Regulation of macrophage and neutrophil cell fates by the PU.1:C/EBPalpha ratio and granulocyte colony-stimulating factor. Nat Immunol. 2003 Oct;4(10):1029–36.

[2] Zhou T, Kinney MC, Scott LM, Zinkel SS, Rebel VI. Revisiting the case for genetically engineered mouse models in human myelodysplastic syndrome research. Blood. 2015 Aug;126(9):1057–68.

[3] Cooper S, Guo H, Friedman AD. The +37 kb Cebpa Enhancer Is Critical for Cebpa Myeloid Gene Expression and Contains Functional Sites that Bind SCL, GATA2, C/EBPÎś, PU.1, and Additional Ets Factors. PLoS One. 2015;10(5):e0126385.

[4] Thorvaldsdóttir H, Robinson JT, Mesirov JP. Integrative Genomics Viewer (IGV): high-performance genomics data visualization and exploration. Brief Bioinform. 2013 Mar;14(2):178–92.

[5] Heinz S, Benner C, Spann N, Bertolino E, Lin YC, Laslo P, et al. Simple combinations of lineage-determining transcription factors prime cis-regulatory elements required for macrophage and B cell identities. Mol Cell. 2010 May;38(4):576–89.

[6] Hamey FK, Nestorowa S, Kinston SJ, Kent DG, Wilson NK, Göttgens B. Reconstructing blood stem cell regulatory network models from single-cell molecular profiles. Proc Natl Acad Sci U S A. 2017 06;114(23):5822–5829.

[7] May G, Soneji S, Tipping AJ, Teles J, McGowan SJ, Wu M, et al. Dynamic analysis of gene expression and genome-wide transcription factor binding during lineage specification of multipotent progenitors. Cell Stem Cell. 2013 Dec;13(6):754–68.

[8] Garber M, Yosef N, Goren A, Raychowdhury R, Thielke A, Guttman M, et al. A high-throughput chromatin immunoprecipitation approach reveals principles of dynamic gene regulation in mammals. Mol Cell. 2012 Sep;47(5):810–22.

[9] Zhao L, Glazov EA, Pattabiraman DR, Al-Owaidi F, Zhang P, Brown MA, et al. Integrated genome-wide chromatin occupancy and expression analyses identify key myeloid pro-differentiation transcription factors repressed by Myb. Nucleic Acids Res. 2011 Jun;39(11):4664–79.

[10] Doré LC, Chlon TM, Brown CD, White KP, Crispino JD. Chromatin occupancy analysis reveals genome-wide GATA factor switching during hematopoiesis. Blood. 2012 Apr;119(16):3724–33.

[11] Chlon TM, Doré LC, Crispino JD. Cofactor-Mediated Restriction of GATA-1 Chromatin Occupancy Coordinates Lineage-Specific Gene Expression. Mol Cell. 2012 Jul;.
